# Impact of rural location on receipt of standard of care treatment and survival for locally advanced bladder cancer in Louisiana

**DOI:** 10.1002/cam4.7301

**Published:** 2024-06-25

**Authors:** Megan Escott, Yong Yi, Ashley Foret, TingTing Li, Mei‐Chin Hsieh, Scott E. Delacroix, Xiao‐Cheng Wu, Mary E. Westerman

**Affiliations:** ^1^ School of Medicine LSU Health Science Center New Orleans Louisiana USA; ^2^ Department of Urology Wake Forest University School of Medicine Winston‐Salem North Carolina USA; ^3^ Louisiana Tumor Registry and Epidemiology New Orleans Louisiana USA; ^4^ School of Public Health LSU Health Science Center New Orleans Louisiana USA; ^5^ Department of Urology LSU Health Science Center New Orleans Louisiana USA

**Keywords:** bladder cancer, healthcare disparities, population health

## Abstract

**Objective:**

We aim to determine the effect of region of residence (urban vs. rural) on the odds of receiving standard of care treatment for locally advanced BCa in Louisiana and its impact on survival outcomes.

**Methods:**

Using the Louisiana Tumor Registry, we identified American Joint Committee on Cancer (AJCC) stage II or III, BCa diagnoses in Louisiana residents between 2010 and 2020. Treatment received was classified as standard or non‐standard of care according to American Urological Association (AUA) guidelines and location of residence was determined using Rural Urban Commuting Area‐Tract‐level 2010 (RUCA). Multivariable logistic regression analyses and multivariate cox proportional hazard analyses were performed.

**Results:**

Of 983 eligible patients, 85.6% (841/983) lived in urban areas. Overall, only 37.5% received standard‐of‐care (SOC) for the definitive management of locally advanced bladder cancer. Individuals living in rural areas (OR 0.53, 95% CI: 0.31–0.91, *p* = 0.02) were less likely to receive standard of care treatment. Both rural residence and receipt of non‐standard of care therapy were associated with an increased risk of bladder cancer‐specific (adj HR 1.53, 95% CI: 1.09–2.14, *p* = 0.01 and adj HR: 1.85, 95% CI: 1.43–2.39, <0.0001) and overall mortality (adj HR: 1.28, 95% CI: 1.01–1.61, *p* = 0.04 and adj HR: 1.73 95% CI: 1.44–2.07, *p* < 0.0001).

**Conclusions:**

Most patients with locally advanced bladder cancer in Louisiana do not receive SOC therapy. Individuals living in rural locations are more likely to receive non‐standard of care therapy than individuals in urban areas. Nonstandard of care treatment and rural residence are both associated with worse survival outcomes for Louisiana residents with locally advanced bladder cancer.

## Graphical abstract

Most patients with locally advanced bladder cancer in Louisiana do not receive standard‐of‐care therapy. Individuals living in rural locations are more likely to receive non‐standard of care therapy than individuals in urban areas. Nonstandard of care treatment and rural residence are both associated with worse survival outcomes for Louisiana residents.

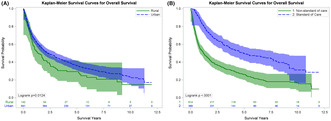

## 1 INTRODUCTION

In 2023, an estimated 81,000 new cases of bladder cancer are projected, with 17,000 anticipated deaths in the United States.^1^ Of these, approximately 30% will present as muscle‐invasive bladder cancer (MIBC).^2^ MIBC represents an advanced stage of disease, making prompt diagnosis and effective management strategies necessary due to the increased risk of metastasis. Treatment for MIBC typically entails a multimodal approach, combining surgical interventions, radiation therapy, and chemotherapy. Consequently, management of MIBC is both time and resource‐intensive, often requiring referral to specialty centers.

Prior research suggests that social and demographic factors significantly contribute to bladder cancer outcomes. Stage at cancer diagnosis is a significant predictor of cancer specific outcomes, with women and African Americans more likely to be diagnosed at advanced stages.^3^ Even when controlling for stage, these groups have worse cancer specific outcomes.^4^, ^5^ Conversely, married men have better cancer outcomes than single men and commercially insured patients have significantly higher survival rates.^6^, ^7^ Therefore, it is reasonable to suspect that a patient's geographic location in conjunction with their demographic factors is likely to impact the care received in the management of MIBC.

Patients in rural health care settings face unique challenges in treating cancers compared to urban areas. These challenges are due to factors such as limited access to specialized cancer centers, a shortage of local healthcare professionals, geographic barriers, and socioeconomic disparities. In cancers of other organs, patients who reside in rural areas have worse cancer specific outcomes. A recent study highlighted that the 5‐year survival rates for several cancers, including lung, breast, prostate, and colorectal cancers were lower for rural residents when compared to their urban counterparts.^8^ This suggests that the unique challenges associated with residency in rural areas likely affect cancer outcomes.

Given that the mean age at bladder cancer diagnosis is 73‐years‐old and patients often have multiple significant medical comorbidities, access to a multidisciplinary treatment team for locally advanced bladder cancer treatment is likely more difficult for individuals in rural locations. We therefore hypothesized that individuals diagnosed with locally advanced (AJCC stage II or III) bladder cancer living in rural Louisiana would have worse cancer specific outcomes than individuals living in urban areas. We secondarily hypothesized that patients living in rural locations are less likely to receive standard of care treatment. Therefore, this study aimed to determine the effect of region of residence (urban vs. rural) on the likelihood of receiving standard of care treatment for locally advanced BCa in Louisiana and its impact on survival outcomes.

## 2 MATERIALS AND METHODS

### 2.1 Data source and population

We identified Louisiana residents diagnosed with AJCC clinical stage II or III bladder cancer between January 1st, 2010 and December 31st, 2020, utilizing data from Louisiana Tumor Registry (LTR) following approval by the Louisiana State University (LSU) Institutional Review Borad (IRB). The LTR is a statewide population‐based registry which participated in both the Surveillance, Epidemiology, and End Results (SEER) Program of the National Cancer Institute and the National Program of Central Cancer Registries (NPCR) of the Centers for Disease Control and Prevention (CDC).^9^ LTR has received gold certification based on data collection standards specified by the North American Association of Central Cancer Registries (NAACCR) every year since 1997. The registry tracks all cancer cases in the state and reporting is mandated by state law within 6 months of diagnosis. All Cases were identified by the International Classification of Disease for Oncology, Third Edition (ICD‐O‐3) site codes C67.0‐C67.9. All cases with a clinical or pathologic diagnosis of AJCC stage II‐III, microscopically confirmed, bladder cancer as the first or only cancer were included. Histology codes 9050–9055 (mesothelioma), 9140 (sarcoma), 9590–9992 (lymphoma/leukemia), death certificate, and autopsy only cases were excluded. In addition, cases with no data to define socioeconomic, race other than non‐Hispanic black (NHB) or non‐Hispanic white (NHW), and age at diagnosis younger than 20 were removed (*n* = 61). We excluded NHB and NHW cases due to small sample size. All eligible patients were followed through December 31, 2020.

Patients were first stratified into cohorts based on their region of residence. We used the Rural Urban Commuting Area‐ Tract‐level 2010 (RUCA) codes to classify census tracts where the patients resided as urban or rural. RUCA codes are based on population density, urbanization, and daily commuting to defined urban cores.^10^ 2010 RUCA definitions were used in which RUCA codes 1.0, 1.1, 2.0, 2.1, 3.0, 4.1, 5.1, 7.1, 8.1 and 10.1 corresponded to urban or metropolitan areas and all other RUCA codes correspond to rural or non‐metropolitan areas.^11^

We separately stratified received treatment as standard or non‐standard of care (nSOC) according to American Urologic Association guidelines for the stage‐dependent treatment of MIBC.^12^ These guidelines endorse radical cystectomy combined with neoadjuvant chemotherapy as the gold standard treatment for patients with localized muscle invasive bladder cancer. Notably, as some groups have argued for a risk‐stratified approach to neoadjuvant chemotherapy in clinical stage II disease, we included cystectomy only as standard of care in this subgroup.^13^ Trimodality therapy incorporating transurethral resection, chemotherapy, and radiation is also a guideline‐concordant option.^12^ The surgical type was classified, based on surgery codes, as transurethral bladder tumor resection (TURBT), partial cystectomy, and radical cystectomy. Surgery considered/no surgery were merged. Data regarding first course of chemotherapy (neoadjuvant or adjuvant) and radiation was also analyzed. Partial cystectomy was not considered standard of care for any patient. For stage III disease, cystectomy without chemotherapy (neoadjuvant or adjuvant) was considered non‐standard of care. Date of death and cause of death was captured and classified according to the SEER cause specific death classification utilizing death certificate data.

#### 2.1.1 Outcomes

Receipt of standard‐of‐care (SOC) treatment for the definitive management of locally advanced bladder cancer was the outcome variable for logistic regression analysis. The bladder cancer specific survival (CSS) and overall survival (OS) mortality were the outcomes for Cox proportional hazard regression analysis.

#### 2.1.2 Exposure

Rural versus urban region of residence.

### 2.2 Covariates

Demographic variables of patients included age at bladder cancer diagnosis (20–59, 60–69, 70–79, ≥80), marital status (single, married, other/unknown), gender (male/female), race (NHW or NHB), insurance status (private, Medicare/other public, Medicaid, no/unknown insurance) at bladder cancer diagnosis and during the first course treatment, and socioeconomic status (SES). SES was compared utilizing YOST‐State based quintiles.^14^ The Yost Index is a socioeconomic index comprising seven variables from the American Community Survey of the United States Census which are frequently used in cancer surveillance.^14^ These variables include median household income, median house value, median rent, percent of population below 150% of poverty line, education index, percent working class, and percent unemployed.^15^ Quintile 1 is defined as lowest SES while quintile 5 is the highest SES.^15^

Clinical features included AJCC stage of disease at the time of diagnosis (stages II or III), and patients' Charlson/Deyo Comorbidity Scores (CCS: based on 16 comorbidities and classified as 0, 1, and 2+ comorbidity scores).^16^ Histology type was defined as transitional (ICD‐O‐38050, 8120–8122, 8130–8131) or other.

### 2.3 Statistical analysis

Descriptive analysis and chi‐squared tests were performed by region of residence (urban vs. rural). Univariate and multivariable logistic regression were performed to assess the impact of urban versus rural residence on receiving standard of care definitive treatment, adjusting for race, gender, age, socioeconomic status, insurance, marital status, AJCC stage, histology type, and charlson comorbidity index. Kaplan–Meier method was used to conduct survival analysis for overall and cancer‐specific survival outcomes. Multivariate Cox proportional hazard analysis was performed to assess the impact of urban versus rural residence and receipt of standard of care therapy on OS and cancer‐specific mortality, adjusting for race, gender, age, socioeconomic status (YOST index), primary payer, marital status, stage, histology, and charlson comorbidity index. The survival period is measured in years starting from the date of cancer diagnosis with follow‐up through December 31, 2021, which is the last date we have good linkage data from our input sources such as state vital statistics and National Death Index. All analyses were performed using SAS statistical software (version 9.4; SAS Institute Inc.). All statistical tests were two‐sided, with a *p* value <0.05 used to identify statistical significance.

## 3 RESULTS

### 3.1 Baseline characteristics

In total 983 patients met inclusion criteria. Baseline characteristics of the cohort are shown in Table 1. Most (*n* = 715, 72.7%) had AJCC stage two disease and lived in a predominately urban setting (*n* = 841, 85.6%). Rural patients were more likely to be male (81% vs. 71%, *p* = 0.03) and to have AJCC stage II disease (80.3% vs. 71.5%). Rural patients were predominately in the lower three SES groups (134/142, 94.3%) compared to urban patients who were mainly in the highest three quartiles (564/841, 67%) (*p* < 0.0001). Overall, 37.5% (*n* = 369) patients received SOC therapy (Table 2), 39.7% of urban residents (323/814) compared to 32.4% of rural residents (46/142), *p* = 0.19. Among those receiving SOC, 19% (70/369) received trimodal therapy. The most common nSOC treatments were TURBT alone (*n* = 280, 45.6%) and TURBT with chemotherapy OR radiation (*n* = 210, 34%). In total 71 (11.5%) patients underwent cystectomy without chemotherapy.

**TABLE 1:** Baseline characteristics and treatment associated features of urban and rural cohorts.

	Total Cohort (*n* = 983)		Urban (*n* = 841)		Rural (*n* = 142)		
	*N*	%	*N*	%	*N*	%	*p*‐value
Race							
Non‐Hispanic white	761	77.4	644	76.6	117	82.4	0.13
Non‐Hispanic black	222	22.6	197	23.4	25	17.6	
Gender							
Male	711	72.3	597	71.0	114	80.3	0.025
Female	272	27.7	244	29.0	28	19.7	
Age							
20–59	178	18.1	155	18.4	23	16.2	0.40
60–69	266	27.1	229	27.2	37	26.1	
70–79	287	29.2	237	28.2	50	35.2	
≥ 80	252	25.6	220	26.2	32	22.5	
Socioeconomic status (YOST index)							
Quintile 1 (lowest SES)	174	17.1	126	15.0	48	33.8	<0.0001
Q2	197	20.0	151	18.0	46	32.4	
Q3	213	21.7	173	20.6	40	28.2	
Q4	190	19.3	182	21.6	8	5.6	
Q5 (highest SES)	209	21.3	209	24.9	0	0.0	
Primary payer							
Private insurance	319	32.5	279	33.2	40	28.2	0.70
Medicare/other public	488	49.6	413	49.1	75	52.8	
Medicaid	144	14.7	122	14.5	22	15.5	
No/unknown insurance	32	3.3	27	3.2	5	3.5	
Marital status							
Single	177	18.0	145	17.2	32	22.5	0.048
Married	497	50.6	420	49.9	77	54.2	
Other/unknown	309	31.4	276	32.8	33	23.2	
AJCC stage							
Stage II	715	72.7	601	71	114	80	0.03
Stage III	268	27.3	240	29	28	20	
Histologic type							
Transitional	908	92.4	776	92	132	93	0.87
Other	75	7.6	65	8	10	7	
Charlson Comorbidity Index							
0	565	57.5	475	56.5	90	63.4	0.23
1	218	22.2	188	22.4	30	21.1	
≥ 2	200	20.4	178	21.2	22	15.5	
Type of surgery							
TURBT only	540	54.9	452	53.7	88	62.0	0.14
Partial cystectomy/surgery, NOS	44	4.5	37	4.4	7	4.9	
Radical cystectomy	371	37.7	325	38.6	46	32.4	
No/unknown surgery	28	2.9	27	3.2	1	0.7	
Chemotherapy							
Yes	455	46.3	393	47	62	44	0.53
No/unknown	528	53.7	448	53	80	56	
Radiation							
Yes	129	13.1%	116	14	13	9	0.14
No/unknown	854	86.9%	725	86	129	91	
Definitive management							
Standard of care	369	37.5	323	38	46	32	0.19
Non‐standard of care	614	62.5	518	62	96	68	

**TABLE 2:** Standard and non‐standard of care classification of treatments for definitive management of locally advanced bladder cancer.

Standard of care (*n* = 369)	Stage II (*n* = 706) *N* (%)	Stage III (*n* = 262) *N* (%)	Total (*n* = 968) *N* (%)
Radical cystectomy and chemotherapy	144 (20.4)	67 (25.6)	211 (21.8)
Radical cystectomy alone (for stage II disease)	88 (12.5)	0	88 (9.1)
TURBT, radiation, and chemotherapy	58 (8.2)	12 (4.6)	70 (7.2)
Non‐standard of care (*n* = 614)			
TURBT alone	236 (33.4)	44 (16.8)	280 (28.9)
TURBT and chemotherapy or radiation (not both)	157 (22.2)	53 (20.2)	210 (21.7)
Radical cystectomy alone for stage III	0	71 (27.1)	71 (7.3)
Partial cystectomy (±chemo/radiation)	16 (2.3)	8 (3.1)	24 (2.5)
Radiation only	2 (0.3)	5 (1.9)	7 (0.7)
Chemotherapy only	5 (0.7)	0	5 (0.5)
Radical cystectomy + radiation	0	1 (0.4)	1 (0.1)
Chemotherapy + radiation (no TURBT)	0	1 (0.4)	1 (0.1)
No/unknown treatment	9 (1.3)	6 (2.3)	15 (1.5)
Total	706 (72.9%)	262 (*n* = 27.1%)	968

### 3.2 Cancer‐specific survival

**FIGURE 1:**
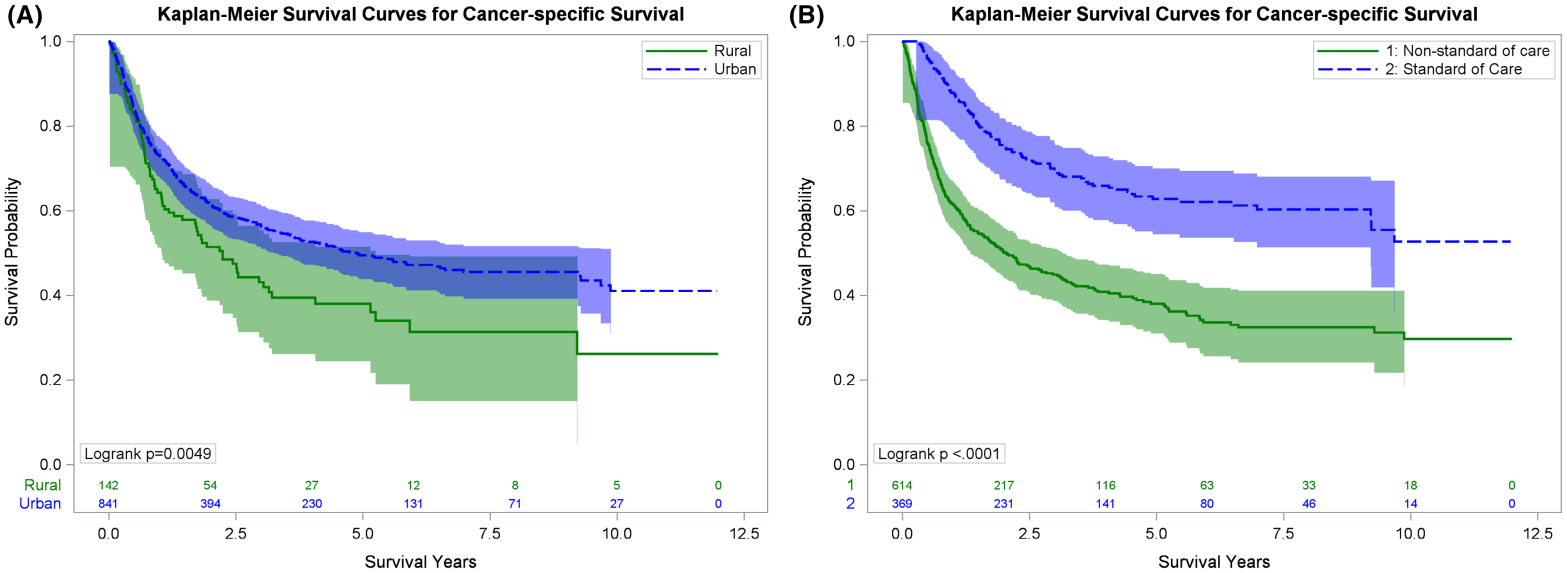
Kaplan–Meier cancer specific survival (CSS) curves for (A) urban and rural residents and (B) receipt of standard of care versus non‐standard of care therapy.

For the entire cohort, the median cancer‐specific survival (CSS) was 51.4 months (95% CI: 37.8–67 months). Residents of urban areas had significantly better CSS—58.7 months (95% CI: 43.9–83.8) compared to 26.9 months (95% CI: 15.4, 37.8) for rural residents (logrank *p* = 0.005) (Figure 1A). Factors associated with cancer‐specific mortality are shown in Table 3. In multivariable hazard analysis, rural residency (HR: 1.53, 95% CI: 1.09, 2.14, *p* = 0.01) as well as nSOC (HR 1.85, 95% CI: 1.43–2.4, *p* < 0.001) were covariates associated with cancer‐specific mortality.

**TABLE 3:** Multivariable analysis of factors associated with cancer specific and overall mortality among Louisiana residents with locally advanced bladder cancer.

	Cancer specific mortality		Overall mortality	
	HR (95% CI)	*p* value	HR (95% CI)	*p* value
Rural commuting areas	1.53 (1.09, 2.14)	0.013	1.28 (1.01,1.61)	0.041
Race				
Non‐Hispanic white (reference)				
Non‐Hispanic black	0.95 (0.70, 1.29)	0.72	0.94 (0.77,1.16)	0.58
Gender				
Female	1.10 (0.84, 1.46)	0.49	1.02 (0.84,1.23)	0.88
Age				
18–59 (reference)				
60–69	0.81 (0.55, 1.20)	0.30	1.09 (0.83, 1.44)	0.52
70–79	1.41 (0.96, 2.07)	0.078	1.44 (1.09,1.91)	0.01
≥ 80	2.33 (1.56, 3.48)	<0.0001	2.54 (1.89,3.40)	< 0.0001
Socioeconomic status (YOST index)				
Quintile 1 (lowest SES) (reference)				
Q2	0.56 (0.39, 0.81)	0.002	0.74 (0.57, 0.95)	0.018
Q3	0.45 (0.31, 0.66)	<0.0001	0.61 (0.47,0.80)	0.000
Q4	0.73 (0.49, 1.07)	0.11	0.75 (0.57, 0.98)	0.036
Q5 (highest SES)	0.65 (0.42, 0.99)	0.044	0.65 (0.49, 0.87)	0.004
Primary payer				
Private insurance (reference)				
Medicare/other public	1.15 (0.86, 1.53)	0.35	1.28 (1.04, 1.56)	0.017
Medicaid	1.64 (1.12, 2.41)	0.011	1.68 (1.29, 2.19)	0.000
No/unknown insurance	1.58 (0.85, 2.95)	0.15	1.34 (0.83, 2.18)	0.24
Marital status				
Single (reference)				
Married (including common law)	0.81 (0.57, 1.16)	0.26	0.87 (0.68, 1.10)	0.24
Other/unknown	0.92 (0.64, 1.33)	0.67	1.02 (0.80, 1.30)	0.87
AJCC stage III	1.65 (1.29, 2.10)	< 0.0001	1.36 (1.14, 1.62)	0.001
Variant histology	1.27 (0.86, 1.88)	0.23	1.11 (0.83, 1.49)	0.47
Charlson comorbidity index				
0 (reference)				
1	1.50 (1.13, 2.00)	0.006	1.19 (0.97, 1.45)	0.098
≥ 2	1.62 (1.20, 2.19)	0.002	1.64 (1.34, 1.99)	< 0.0001
Non‐standard of care management	1.85 (1.43, 2.39)	<0.0001	1.73 (1.44, 2.07)	<0.0001

### 3.3 Overall survival

**FIGURE 2:**
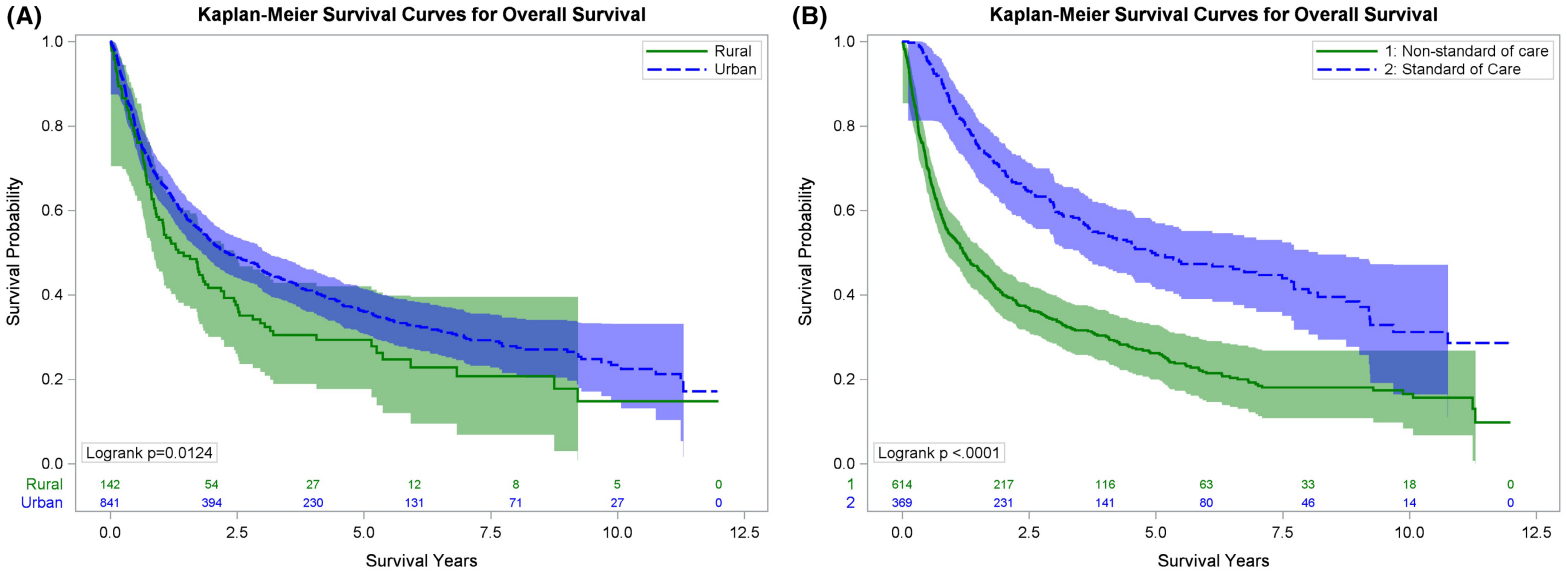
Kaplan–Meier overall survival (OS) curves for (A) urban and rural residents and (B) receipt of standard of care versus non‐standard of care therapy.

For the entire cohort, the median OS was 25 months, (95% CI: 21.8–30.6 months)—27.7 months (95% CI 23.2–35.7 months) for urban residents compared to 16.1 months (95% CI 11.0–23.2 months) for residents of rural areas (logrank *p* = 0.01) (Figure 2A). In multivariable hazard analysis, rural residency (HR 1.28, 95% CI: 1.0–1.6, *p* = 0.04) and nSOC treatment (HR 1.73, 95% CI: 1.4–2.1, *p* < 0.0001) were covariates associated with mortality.

### 3.4 Receipt of standard of care therapy

We then separately analyzed the impact of receipt of SOC versus nSOC on CSS and OS (Figures 1B and 2B). The median CSS was not reached for patients receiving SOC compared to 24.5 months (95% CI: 19.1–32.9) for patients receiving nSOC (*p* < 0.0001) (Figure 1B). Similar differences were seen with OS—those receiving SOC had a median OS of 59.1 months (95% CI: 44.9–89.9) compared to 14.5 months (95% CI: 11.7–18.0 months) for patients receiving nSOC (*p* < 0.0001) (Figure 2B).

Finally, we looked the association of region of residence (urban vs. rural) with receipt of nSOC therapy. Table 4 shows the multivariable logistic regression analysis on the odds ratios of receiving SOC. After adjusting for covariates, rural residents were less likely to receive standard of care treatment (OR: 0.53, 95% CI: 0.31–0.91, *p* = 0.21).

**TABLE 4:** Results of multivariate logistic analysis on the odds ratios of receiving standard of care.

Odds ratio estimates and wald confidence intervals				
Effect	Estimate	95% confidence limits		*p*‐value
Region: rural versus urban	0.53	0.31	0.91	0.02
Race: NH Black versus NH White	0.60	0.37	0.95	0.03
Gender: female versus male	0.90	0.60	1.36	0.63
Age 60–69 versus 20–59	0.78	0.47	1.32	0.36
Age 70–79 versus 20–59	0.70	0.40	1.21	0.2
Age 80+ versus 20–59	0.19	0.10	0.35	<0.0001
SES Group 2 versus Group 1 (lowest SES)	0.88	0.49	1.58	0.66
SES Group 3 versus Group 1 (lowest SES)	1.05	0.58	1.91	0.87
SES Group 4 versus Group 1 (lowest SES)	0.99	0.52	1.86	0.97
SES Group 5 (highest SES) versus Group 1 (lowest SES)	0.80	0.42	1.52	0.5
Insurance medicare/other public versus private insurance	0.95	0.63	1.42	0.8
Insurance medicaid versus private insurance	0.56	0.31	1.01	0.052
Insurance no/unknown insurance versus private insurance	0.73	0.31	1.71	0.5
Marital status: married versus single	1.06	0.62	1.84	0.82
Marital status: other/unknown versus single	1.07	0.60	1.89	0.82
AJCC stage 3 versus 2	0.46	0.31	0.69	<0.0001
Histology type: other versus transitional	0.79	0.41	1.53	0.49
CCI 1 versus 0	1.16	0.76	1.78	0.49
CCI ≥2 versus 0	0.67	0.42	1.08	0.1

## 4 DISCUSSION

In this statewide population‐based analysis, we sought to understand if rural residence was associated with worse survival outcomes and if this was attributable to receiving nSOC therapy. We found that overall, only 37.5% of individuals received SOC treatment for locally advanced bladder cancer. Importantly, individuals living in rural Louisiana were less likely to receive SOC treatment. Individuals who received nSOC treatment as well as individuals who live in rural areas have worse survival outcomes than those living in urban areas or receiving SOC. Notably, while Black individuals were less likely to receive SOC treatment. When adjusting for other variables including SES and type of insurance coverage, race did not impact survival outcomes.

Our findings align with previous literature suggesting that rural residency may independently impact both overall and bladder cancer‐specific survival. In our sample population, rural residency was associated with decreased odds of receiving SOC treatment. However, nSOC treatment does not fully explain the disparity in outcomes between urban and rural residents as region of residence remained strongly associated with significantly greater overall mortality and cancer‐specific mortality even after controlling for type of treatment.

Socioeconomic status (SES) and insurance coverage have been investigated in attempts to understand the disparities seen between urban and rural populations. Lower SES may hinder access to regular healthcare, delay time to treatment and decrease the ability to follow‐up postoperatively, all of which have been associated with worse outcomes.^17^ However, the recent literature is mixed on the impact of these factors. For example, two studies utilizing statewide databases (California and Pennsylvania) found that SES and insurance coverage were associated with decreased cause specific survival rather than of rurality of residence alone.^18^, ^19^ Conversely, an analysis of national census data found that rural patients had significantly higher overall mortality and marginally increased cancer‐specific mortality despite controlling for SES and insurance coverage.^20^

We found that in Louisiana, rural residence, SES, and insurance status all impact survival outcomes, despite adjustment for receipt of SOC treatment. While Louisiana has one of the highest proportions of Medicaid insured population nationally (25.2%–37.1%), only 14.7% of patients in the study had Medicaid.^21^ Likewise, 3.3% of the cohort was uninsured, compared to 7.6% of the state‐wide population.^22^ These differences are likely attributable to the age of the population and reflect the eligibility of Medicare eligibility after age 65, which is independent of SES. In addition, the Medicaid expansion was implemented in July 2017, whereas this study included bladder cancer cases diagnosed in 2010–2020, which may also contribute to the lower‐than‐expected Medicaid population. We found that neither SES or insurance coverage impacted the odds of receiving SOC treatment, however both were associated with survival outcomes, with patients on Medicaid and those in the lowest SES group having worse OS and CSS.

It has also been hypothesized that difficulty in accessing adequate treatment often found in rural areas could explain decreased survival in rural patients. Gore et al found that an increased travel distance, which is often the situation faced by rural residents, resulted in decreased odds of receiving a radical cystectomy in a national dataset.^23^ Notably this study utilized SEER‐Medicare linkage data and was limited to individuals diagnosed with cancer after age 66. In addition, when analyzing travel distance on a state level, a different study found that an increased distance needed to undergo cystectomy did not predict likelihood of receiving treatment or increased risk of hospital readmissions.^24^ In Louisiana, tertiary referral centers are limited to the Gulf Coast and northwestern border with Texas. Time to definitive treatment as well as intensity of follow up may be influenced by travel distance and access to care.

While the aim of our study was not to conduct an exploratory analysis of risk factors for nSOC treatment, it is worth noting that Black individuals were less likely to receive SOC treatment. With that in mind, when adjusting for other variables including SES and type of insurance coverage, race did not impact survival outcomes. Our cohort is unique in that black patients comprise 22.6% of our population, which is significantly larger than most published observational data sets. ^25^ Previous studies have found that Black individuals have the highest risk of bladder cancer death, as much as 70% higher than whites, which has been hypothesized to be related to SES, increased tobacco use, and/or occupational exposures.^26^, ^27^, ^28^ The lack of association of race with CSS aligns with recent findings from Freudenburg et al., suggesting that the differences observed in bladder cancer specific mortality are probably a result of healthcare disparities and demographic variations specific to certain geographical areas, rather than solely attributable to racial disparities.^29^ We hypothesize that observed racial differences in bladder cancer survival outcomes are related to the ability to access to standard of care treatments rather than biologic differences and future studies improving access to SOC treatments is needed.

While rural and black individuals (as well as older patients and those with AJCC stage III disease) are more likely to receive nSOC treatment, it is important to note that less than 40% of patients with MIBC across the state receive SOC treatment. This is consistent with previous evaluations of guideline‐concordant care delivery in non‐muscle invasive bladder cancer (NMIBC) where less than 30% of patients in Europe and North America receive SOC based on accepted guidelines.^15^, ^16^ Similarly, in a 2017 SEER analysis of patients diagnosed with MIBC between 2007 and 2013, Williams et al. found that only 6% of patients with MIBC underwent RC.^30^

We utilized the RUCA classification system combines population size, commuting data, and proximity to urbanized area rather than the US Census Bureau definition. Compared to the US Census Bureau definitions of urban and rural areas, the RUCA more accurately reflects the US population. In 2010, 19.3% of patients resided in a rural area, but only 1.6% of the SEER population with a bladder cancer diagnosis lived in a rural area in a recent analysis utilizing US Census definitions.^31^ Using the RUCA classification, 14.5% of our patients were residents of rural areas, which is more reflective of the state‐wide population, which is approximately 20% rural. In their SEER analysis, Deuker et al. concluded that the database does not reflect the US in terms of urban versus rural residency status areas.^20^ However, while the LTR is one of the SEER registries, per Louisiana legislative rules (LAC 48:V.8501–8513) all Louisiana healthcare facilities are required to abstract and report each case of cancer to the LTR within 6 months of diagnosis, thus all cases within the state (rather than a region) are captured.

The strength of our study is that it is a population‐based study with detailed patient and tumor characteristics. However, there are limitations to our study. Our results may be biased by unmeasured confounding or incomplete adjustment. In addition, our data is limited to Louisiana cancer cases only and findings may not be applicable to other geographic regions. However, based on our findings and in the context of recent work, we believe that healthcare disparities are best studied at a local and regional level as geographic variations, local politics, and access to care vary widely across regions. Attempting to draw broad conclusions based on large national datasets may result in oversimplification. In addition, we defined standard of care based on AJCC stage. Pathologic stage was prioritized when available, otherwise clinical stage was used. Inaccurate clinical staging or incomplete chemotherapy or radiation data may lead to incorrect classification of treatment as standard or non‐standard of care. In addition, we cannot stratify patients by platinum eligibility, so a subset of patients with AJCC stage III disease ineligible for platinum‐based chemotherapy who received cystectomy only may be incorrectly classified as receiving “non‐standard or care therapy.” For trimodal therapy, granular details regarding radiation dose (palliative vs. definitive) and type and sequencing of radio sensitizing chemotherapy were not analyzed. However, by only classifying patients who got both chemotherapy and radiation without radical cystectomy as receiving SOC, those treated with palliative intent radiotherapy would be captured as nSOC. In addition, the data on adjuvant chemo and radiation therapy in the LTR database may not be 100% complete.

Individuals may have traveled out of state to receive definitive treatment. However, the LTR has interstate data exchange agreements with 50 population‐based cancer registries, including 45 states (all neighboring states), Washington D.C., three United States territories (Guam, Puerto Rico, and the Virgin Islands), and Bermuda. Data exchange is done twice a year; therefore, data is obtained for patients who traveled out of state to receive MIBC care. Finally, we analyzed SES using the Yost State‐based quintile.^14^, ^15^ These quintiles are state specific and therefore comparisons may not be applicable to other states.

## 5 CONCLUSION

Most patients with locally advanced bladder cancer in Louisiana do not receive standard of care therapy. Rural individuals are more likely to receive non‐standard of care therapy than urban individuals. Nonstandard of care treatment and rural residence, are both associated with worse survival outcomes for Louisiana residents with locally advanced bladder cancer. Further work to improve access to standard of care treatment is needed, with particular focus on rural residents.

## AUTHOR CONTRIBUTIONS

**Megan Escott:** Writing – original draft (lead). **Yong Yi:** Data curation (lead); formal analysis (lead). **Ashley Foret:** Writing – original draft (equal); writing – review and editing (equal). **TingTing Li:** Data curation (equal); formal analysis (equal). **Mei‐Chin Hsieh:** Writing – review and editing (equal). **Scott E. Delacroix:** Writing – review and editing (equal). **Xiao‐Cheng Wu:** Writing – review and editing (equal). **Mary E. Westerman:** Conceptualization (lead); data curation (equal); supervision (lead); writing – original draft (equal); writing – review and editing (lead).

## FUNDING INFORMATION

National Cancer Institute (HHSN261201800007I/HHSN26100002) to the LSU Health New Orleans for the Surveillance, Epidemiology, End results Program. Centers for Disease Control and Prevention (NU58DP006332) to the LSU Health New Orleans for the National Program of Cancer Registries.

## CONFLICT OF INTEREST STATEMENT

The authors have no conflict of interest to declare.

## ETHICS STATEMENT

This research project, titled “Impact of Rural Location on Receipt of Standard of Care Treatment and Survival for Locally Advanced Bladder Cancer in Louisiana,” falls under the exempt category as defined by the Louisiana State University Health Science Center (LSUHSC) Institutional Review Board (IRB) guidelines (IRB #6315). The exempt status is granted based on the minimal risk and specific criteria outlined in the regulations governing exempt research. The purpose of this ethics statement is to highlight the ethical considerations specific to this exempt research. Informed consent procedures were not required for this exempt research, as the study involves minimal risk to participants, and the data collected is either anonymous or cannot be linked to individuals. Participants were not subjected to any interventions or interactions, and their participation was voluntary.

## PRECIS

Most patients with locally advanced bladder cancer in Louisiana do not receive standard of care therapy. Rural and Black residents are more likely to receive non‐standard of care therapy, while nonstandard of care treatment and rural residence are associated with worse survival outcomes.

## Supporting information


Data S1:

## Data Availability

Data are available from the authors with the permission of Louisiana Tumor Registry. The data that support the findings of this study are available from the corresponding author, MEW, upon reasonable request.

## References

[1] Siegel RL , Miller KD , Fuchs HE , Jemal A . Cancer statistics, 2022. CA Cancer J Clin. 2022;72(1):7‐33. doi:10.3322/caac.21708 35020204

[2] Miller KD , Nogueira L , Devasia T , et al. Cancer treatment and survivorship statistics, 2022. CA Cancer J Clin. 2022;72(5):409‐436. doi:10.3322/caac.21731 35736631

[3] Weiner AB , Keeter MK , Manjunath A , Meeks JJ . Discrepancies in staging, treatment, and delays to treatment may explain disparities in bladder cancer outcomes: an update from the National Cancer Data Base (2004–2013). Urol Oncol. 2018;36(5):237.e9‐237.e17. doi:10.1016/j.urolonc.2017.12.015 29338913

[4] Marinaro J , Zeymo A , Egan J , et al. Sex and racial disparities in the treatment and outcomes of muscle‐invasive bladder cancer. Urology. 2021;151:154‐162. doi:10.1016/j.urology.2020.06.087 32810481

[5] Scosyrev E , Noyes K , Feng C , Messing E . Sex and racial differences in bladder cancer presentation and mortality in the US. Cancer. 2009;115(1):68‐74. doi:10.1002/cncr.23986 19072984

[6] Niu Q , Lu Y , Wu Y , et al. The effect of marital status on the survival of patients with bladder urothelial carcinoma: a SEER database analysis. Medicine. 2018;97(29):e11378. doi:10.1097/MD.0000000000011378 30024509 PMC6086512

[7] Fletcher SA , Cole AP , Lu C , et al. The impact of underinsurance on bladder cancer diagnosis, survival, and care delivery for individuals under the age of 65 years. Cancer. 2020;126(3):496‐505. doi:10.1002/cncr.32562 31626340

[8] Lewis‐Thames MW , Langston ME , Khan S , et al. Racial and ethnic differences in rural‐urban trends in 5‐year survival of patients with lung, prostate, breast, and colorectal cancers: 1975–2011 Surveillance, Epidemiology, and End Results (SEER). JAMA Netw Open. 2022;5(5):e2212246. doi:10.1001/jamanetworkopen.2022.12246 35587350 PMC9121191

[9] Louisiana Tumor Registry: About the Registry . 2023. Accessed October 16, 2023. https://sph.lsuhsc.edu/louisiana‐tumor‐registry/about‐the‐registry/.

[10] Moss JL , Stinchcomb DG , Yu M . Providing higher resolution indicators of rurality in the Surveillance, Epidemiology, and End Results (SEER) database: implications for patient privacy and research. Cancer Epidemiol Biomarkers Prev. 2019;28(9):1409‐1416. doi:10.1158/1055-9965.EPI-19-0021 31201223 PMC6726549

[11] Urban and Rural. United States Census Bureau . Published September 26, 2023. Accessed October 16, 2023. https://www.census.gov/programs‐surveys/geography/guidance/geo‐areas/urban‐rural.html.

[12] Chang SS , Bochner BH , Chou R , et al. Treatment of non‐metastatic muscle‐invasive bladder cancer: AUA/ASCO/ASTRO/SUO guideline. J Urol. 2017;198(3):552‐559. doi:10.1016/j.juro.2017.04.086 28456635 PMC5626446

[13] Culp SH , Dickstein RJ , Grossman HB , et al. Refining patient selection for neoadjuvant chemotherapy before radical cystectomy. J Urol. 2014;191(1):40‐47. doi:10.1016/j.juro.2013.07.061 23911605 PMC4158919

[14] Yu M , Tatalovich Z , Gibson JT , Cronin KA . Using a composite index of socioeconomic status to investigate health disparities while protecting the confidentiality of cancer registry data. Cancer Causes Control. 2014;25(1):81‐92. doi:10.1007/s10552-013-0310-1 24178398

[15] Yost K , Perkins C , Cohen R , Morris C , Wright W . Socioeconomic status and breast cancer incidence in California for different race/ethnic groups. Cancer Causes Control. 2001;12(8):703‐711. doi:10.1023/a:1011240019516 11562110

[16] Charlson M . Charlson Comorbidity Index (CCI). https://www.mdcalc.com/calc/3917/charlson‐comorbidity‐index‐cci

[17] Hollenbeck BK , Dunn RL , Ye Z , Hollingsworth JM , Lee CT , Birkmeyer JD . Racial differences in treatment and outcomes among patients with early stage bladder cancer. Cancer. 2010;116(1):50‐56. doi:10.1002/cncr.24701 19877112 PMC2807891

[18] Shah AA , Sun Z , Eom KY , et al. Treatment disparities in muscle‐invasive bladder cancer: evidence from a large statewide cancer registry. Urol Oncol. 2022;40(4):164.e17‐164.e23. doi:10.1016/j.urolonc.2021.12.004 35022140

[19] Sung JM , Martin JW , Jefferson FA , et al. Racial and socioeconomic disparities in bladder cancer survival: analysis of the California Cancer Registry. Clin Genitourin Cancer. 2019;17(5):e995‐e1002. doi:10.1016/j.clgc.2019.05.008 31239240 PMC7821748

[20] Deuker M , Stolzenbach LF , Collà Ruvolo C , et al. Bladder cancer stage and mortality: urban vs. rural residency. Cancer Causes Control. 2021;32(2):139‐145. doi:10.1007/s10552-020-01366-1 33230694 PMC7810614

[21] Percentage of Population Enrolled in Medicaid or CHIP by State . https://www.medicaid.gov/state‐overviews/scorecard/percentage‐of‐population‐enrolled‐medicaid‐or‐chip‐state/index.html.

[22] Uninsured in United States . America's Health Rankings. Accessed October 16, 2023. https://www.americashealthrankings.org/explore/measures/HealthInsurance

[23] Gore JL , Litwin MS , Lai J , et al. Use of radical cystectomy for patients with invasive bladder cancer. J Natl Cancer Inst. 2010;102(11):802‐811. doi:10.1093/jnci/djq121 20400716 PMC3245689

[24] Smith AB , Meyer AM , Meng K , et al. The relationship of travel distance with cystectomy access and outcomes. Urol Oncol. 2018;36(6):308. e1‐308. e9. doi:10.1016/j.urolonc.2018.03.005 29566978

[25] Shu TD , Schumacher FR , Conroy B , et al. Disparities in cause‐specific mortality by race and sex among bladder cancer patients from the SEER database. Cancer Causes Control. 2023;34(6):521‐531. doi:10.1007/s10552-023-01679-x 36882598

[26] Prout GR , Wesley MN , McCarron PG , et al. Survival experience of black patients and white patients with bladder carcinoma. Cancer. 2004;100(3):621‐630. doi:10.1002/cncr.11942 14745881

[27] Barki C , Rahmouni HB , Labidi S . The impact of socioeconomic variables status on bladder cancer treatment outcomes during the COVID‐19 pandemic. OAlib. 2021;8(9):1‐22. doi:10.4236/oalib.1107921

[28] Liu Y , Zhao YC , Lu Y , Goodarz D , Gershman B . The role of smoking in explaining racial/ethnic disparities in bladder cancer incidence in the United States. Urol Oncol. 2023;41(9):389. e1‐389. e6. doi:10.1016/j.urolonc.2023.01.025 36849327

[29] Freudenburg E , Shan Y , Martinez A , et al. Geographic distribution of racial differences in mortality in muscle‐invasive bladder cancer patients: an opportunity for improvement. Cancer Causes Control. 2022;33(4):613‐622. doi:10.1007/s10552-022-01553-2 35050417

[30] Williams SB , Huo J , Kosarek CD , et al. Population‐based assessment of racial/ethnic differences in utilization of radical cystectomy for patients diagnosed with bladder cancer. Cancer Causes Control. 2017;28(7):755‐766. doi:10.1007/s10552-017-0902-2 28477210 PMC5497706

[31] Ratcliffe M , Burd C , Holder K , Fields A . Defining Rural at the U.S. Census Bureau. Accessed October 16, 2023. https://www2.census.gov/geo/pdfs/reference/ua/Defining_Rural.pdf. Retrieved 2nd of April 2020.

