# Supplementary material for: Impact of rural location on receipt of standard of care treatment and survival for locally advanced bladder cancer in Louisiana
Source: Cancer Med. 2024 Jun 25;13(12):e7301. doi: 10.1002/cam4.7301 (PMC11199337; doi:10.1002/cam4.7301)
Supplement: Supplementary file 1 — Data S1: [file CAM4-13-e7301-s001.docx]

Supplemental Table 1: Unadjusted Cancer Specific and Overall Survival Models among Louisiana Residents with locally advanced bladder cancer

|  | Cancer Specific Mortality | | Overall Mortality | |
| --- | --- | --- | --- | --- |
|  | HR (95%CI) | P Value | HR (95%CI) | P Value |
| **Rural commuting areas** | **1.60 (1.14, 2.24)** | **0.007** | **1.47 (1.10, 1.96)** | **0.010** |
| **Race** |  |  |  |  |
| Non-Hispanic white (reference) |  |  |  |  |
| Non-Hispanic black | **1.17 (0.87, 1.56)** | **0.301** | **1.08 (0.85, 1.38)** | **0.533** |
| Other | **0.94 (0.35, 2.53)** | **0.900** | **0.96 (0.43, 2.15)** | **0.912** |
| **Female Gender** | **1.28 (0.97, 1.68)** | **0.087** | **1.13 (0.89, 1.42)** | **0.314** |
| **Age** |  |  |  |  |
| 18-59 (reference) |  |  |  |  |
| 60-69 | **0.78 (0.49, 1.23)** | **0.277** | **1.01 (0.68, 1.50)** | **0.954** |
| 70-79 | **1.11 (0.72, 1.69)** | **0.638** | **1.40 (0.96, 2.03)** | **0.083** |
| ≥ 80 | **2.00 (1.33, 3.00)** | **0.001** | **2.67 (1.86, 3.83)** | **<0.001** |
| **Socioeconomic status (YOST index)** |  |  |  |  |
| Quintile 1 (lowest SES) (reference) |  |  |  |  |
| Q2 | **0.93 (0.64, 1.35)** | **0.707** | **0.84 (0.61, 1.17)** | **0.311** |
| Q3 | **0.61 (0.41, 0.91)** | **0.016** | **0.75 (0.54, 1.04)** | **0.084** |
| Q4 | **0.74 (0.51, 1.09)** | **0.131** | **0.83 (0.6, 1.14)** | **0.251** |
| Q5 (highest SES) | **0.54 (0.36, 0.81)** | **0.003** | **0.73 (0.53, 1.01)** | **0.056** |
| **Primary Payer** |  |  |  |  |
| Private Insurance (reference) |  |  |  |  |
| Medicaid | **0.67 (0.28, 1.62)** | **0.376** | **0.75 (0.35, 1.62)** | **0.464** |
| Other Government | **1.02 (0.50, 2.08)** | **0.953** | **1.12 (0.64, 2.28)** | **0.552** |
| Unknown | **0.52 (0.24, 1.12)** | **0.094** | **0.59 (0.30, 1.17)** | **0.129** |
| Not Insured | **1.41 (0.53, 3.76)** | **0.491** | **1.07 (0.42, 2.71)** | **0.888** |
| **Marital Status** |  |  |  |  |
| Single (reference) |  |  |  |  |
| Married (including common law) | **0.84 (0.58, 1.22)** | **0.347** | **0.87 (0.63, 1.19)** | **0.387** |
| Other | **1.16 (0.78, 1.72)** | **0.466** | **1.30 (0.94, 1.81)** | **0.118** |
| Unknown | **0.62 (0.31, 1.21)** | **0.160** | **0.78 (0.47, 1.32)** | **0.356** |
| **AJCC stage III** | **1.53 (1.16, 2.0)** | **0.002** | **1.21 (0.96, 1.53)** | **0.106** |
| **Variant Histology** | **1.47 (0.98, 2.20)** | **0.065** | **1.25 (0.88, 1.78)** | **0.213** |
| **Charlson Comorbidity Index** |  |  |  |  |
| 0 (reference) |  |  |  |  |
| 1 | **1.14 (0.81, 1.59)** | **0.461** | **1.31 (1.01, 1.71)** | **0.043** |
| ≥ 2 | **1.71 (1.25, 2.33)** | **0.001** | **1.70 (1.31, 2.20)** | **<0.0001** |
| **Non-Standard of Care Management** | **2.69 (1.98, 3.42)** | **<0.0001** | **2.42 (1.94, 3.01)** | **<0.0001** |
